# Assessment and management of fluid overload in children on dialysis

**DOI:** 10.1007/s00467-018-3916-4

**Published:** 2018-03-09

**Authors:** Wesley Hayes, Fabio Paglialonga

**Affiliations:** 1 Great Ormond Street Hospital, London, UK; 2 University College London Institute of Child Health, London, UK; 3 Pediatric Nephrology, Dialysis and Transplant Unit, Fondazione IRCCS Ca’ Granda Ospedale Maggiore Policlinico, Milan, Italy

**Keywords:** Dialysis, Fluid balance, Hypertension, Children, Ultrasonography

## Abstract

Dysregulation of intravascular fluid leads to chronic volume overload in children with end-stage kidney disease (ESKD). Sequelae include left ventricular hypertrophy and remodeling and impaired cardiac function. As a result, cardiovascular complications are the commonest cause of mortality in the pediatric dialysis population. The clinical need to optimize intravascular volume in children with ESKD is clear; however, its assessment and management is the most challenging aspect of the pediatric dialysis prescription. Minimizing chronic fluid overload is a key priority; however, excessive ultrafiltration is toxic to the myocardium and can precipitate intradialytic symptoms. This review outlines emerging objective techniques to enhance the assessment of fluid overload in children on dialysis and outlines evidence for current management strategies to address this clinical problem.

## Introduction

Dysregulation of intravascular fluid volume contributes to cardiovascular morbidity and ultimately mortality in children with end-stage kidney disease (ESKD). Cardiovascular complications are the most common cause of mortality in the pediatric dialysis population [1]. Chronic intravascular volume overload is a major risk factor for cardiovascular complications such as left ventricular hypertrophy in children [2, 3]. In adult dialysis patients, chronic volume overload is clearly associated with increased mortality [4]. It is imperative that children’s dialysis prescriptions include sufficient dialysis frequency and ultrafiltration to minimize chronic fluid overload. Conversely, excessive ultrafiltration is toxic to children’s myocardium [5] and can precipitate intradialytic symptoms [6, 7].

The clinical need to optimize intravascular volume in children with ESKD is clear; however, its assessment and management is the most challenging aspect of the pediatric dialysis prescription. Physical examination is notoriously unreliable in the clinical assessment of fluid status. Children frequently experience adverse intradialytic symptoms related to ultrafiltration [6, 8]. There is often a tradeoff between achieving euvolemia and minimizing hemodynamic instability and intradialytic symptoms [9].

This review outlines emerging objective techniques to enhance the assessment of fluid overload in children on dialysis and outlines evidence for current management strategies to address this clinical problem.

## Assessment of fluid overload

Excess fluid in children with ESKD is distributed between the intravascular and interstitial compartments in steady-state conditions; the clinically relevant parameter is intravascular fluid overload as this directly influences cardiac output, systemic blood pressure and cardiovascular sequelae.

**Fig. 1:**
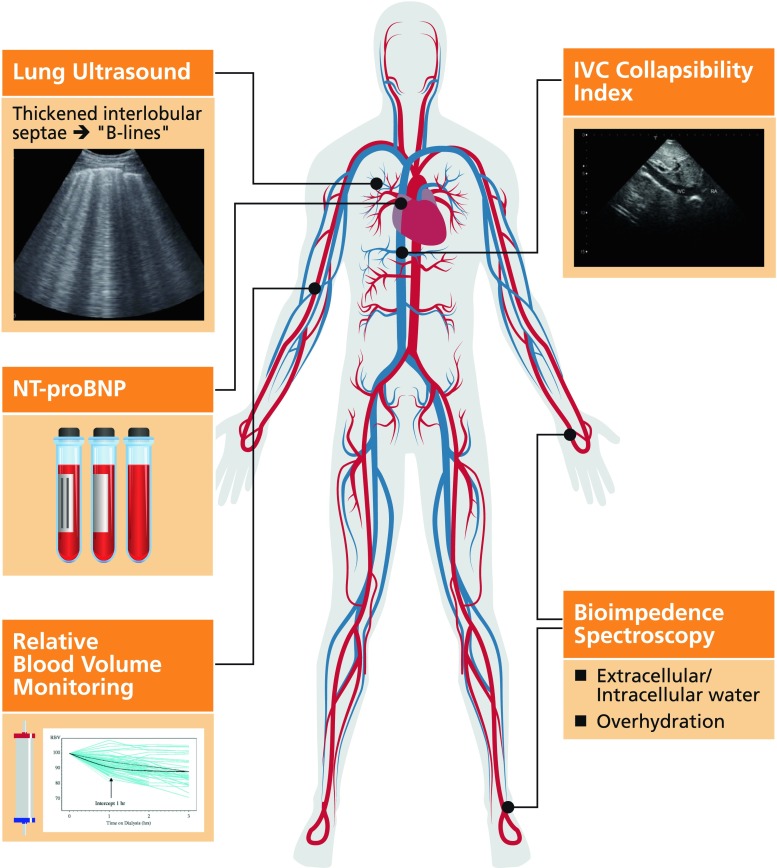
Summary of objective techniques to enhance clinical assessment of fluid overload. *NT-Pro BNP* N-terminal pro-brain natriuetic peptide, *IVC* inferior vena cava

Regular physical examination together with assessment of pre-dialytic blood pressure and inter-dialytic weight gain (IDWG) are currently the main stay of fluid assessment in pediatric dialysis patients [3]. However, current clinical assessments are not sufficient to optimize the target weight in children receiving dialysis for a number of reasons. Firstly, physical examination is insensitive to fluid overload until the level approaches 10% of the child’s bodyweight, which represents severe fluid overload. Secondly, pre-dialytic blood pressure is not solely determined by intravascular volume and is confounded by factors such as impaired cardiac function and physiological variation with exercise and stress. Thirdly, in the pediatric dialysis population, a proportion of interdialytic weight gain should constitute nutritional weight gain, which can be challenging to delineate from fluid overload. This is particularly pertinent for infants and young children, in whom nutritional weight gain can represent 5–10% change in target weight per week. For these reasons, objective measures of fluid overload are needed to enhance regular clinical assessments. A number of emerging techniques to facilitate objective measurement of fluid overload in pediatric dialysis patients are summarized in Fig. 1 and will now be discussed.

### Bioimpedance analysis (BIA)

Bioimpedance spectroscopy (BIS) is a technique used to estimate total body water, extracellular water, lean and adipose tissue mass and overhydration (OH). Various devices are available; however, the Fresenius body composition monitor (Fresenius Medical Care, 61,352 Bad Homburg, Germany) is the most widely used in dialysis patients. This device estimates hydration parameters from 50 different alternating current frequencies between adhesive electrodes on the placed on the hand and wrist and foot and ankle.

Limited data are available on the accuracy of BIS in quantifying fluid overload in children with ESKD. In a small study comparing BIS to deuterium and bromide dilution measurements in 16 children, BIS was not found to accurately estimate total body water or extracellular water volume, albeit with significant study limitations such as non-supervised ingestion of deuterated water in patients’ homes [10]. Overhydration assessed using BIS was compared to systolic blood pressure in a retrospective analysis of 23 children; no correlation was found [11]. A key limitation of this study was that blood pressure was not normalized to account for patient height. This issue was addressed in a study of 30 children with chronic kidney disease (CKD) and 13 controls, but again found no correlation between over-hydration measurements from BIS and systolic blood pressure *Z* score in children [12].

Indexed left ventricular mass index (LVMI) and the incidence of pulmonary edema were compared between 18 teenage dialysis patients (median 14.8 years) after the introduction of single-frequency BIA, to data from 13 patients (median age 15.6 years) in whom BIA was not used. Improvements in LVMI and pulmonary edema were reported following the introduction of BIA to guide target weight [13].

In summary, there are currently no data to support the use of BIA to quantify fluid overload in the pediatric dialysis population. This technique may however have a role in guiding assessment of target weight by evaluating trends in individual patients.

### Inferior vena cava collapsibility index

Echocardiographic assessment of inferior vena cava (IVC) parameters was proposed as a tool to estimate target weight for adults on dialysis nearly 2 decades ago [14] and has subsequently been confirmed to reflect excess intravascular volume [15]. Pediatric data on this technique are sparse, with one study demonstrating improvement in IVC collapsibility index following ultrafiltration in 16 children on peritoneal dialysis (PD) and 9 on hemodialysis (HD) [16].

This technique has a number of limitations. Firstly, it is not suitable for infants, young children or those who are unable to cooperate with holding their breath on demand. Secondly, it is challenging to obtain adequate images even for highly trained observers, so not entirely practical as a bedside tool. These issues may underlie the lack of widespread uptake of this method in clinical practice.

### Relative blood volume monitoring

Relative blood volume transducers are a feature of most modern hemodialysis machines. This technique allows real-time assessment of the relative change in hematocrit or protein concentration in response to ultrafiltration during hemodialysis [17]. Relative blood volume decreases with ultrafiltration with the slope of the curve determined by the rate of fluid removal.

Studies on adult patients have reported benefits of this technique in reducing symptomatic hypotension [18] and aiding the assessment of target weight [19]. A recent well-conducted randomized trial of blood volume-guided ultrafiltration via biofeedback, which automatically adjusts the ultrafiltration rate according to blood volume parameters, did not show any benefit in reducing symptomatic intradialytic hypotension [20].

Limited pediatric data are available on this technique. The slope of the relative blood volume curve in the first hour of hemodialysis was found to predict intradialytic symptoms in a single-centre pediatric study [7]. There are currently no studies to support routine use of this technique in the assessment of target weight in children on dialysis.

### N-terminal pro-brain natriuretic peptide (NT-proBNP)

NT-proBNP is a natriuretic peptide used as a biomarker to aid the diagnosis of cardiac failure in adult patients. It has also been shown to predict cardiac dysfunction in children and adolescents [21]. NT-proBNP is associated with mortality in adults with ESKD [22], and pre-dialysis levels correlate with extracellular volume excess assessed by bioimpedance spectroscopy in this population [23]. There are currently no studies evaluating the utility of this biomarker in the identification of intravascular volume overload in children on dialysis.

### Lung ultrasound

Lung ultrasound is increasingly used in adults on hemo- and peritoneal dialysis to detect extravascular lung water as a marker of generalized fluid overload [24–27]. This technique detects thickening of interlobular lung septae found in subclinical fluid overload [28, 29]. It is significantly more sensitive than auscultation or chest X-ray [30].

The physiological principle underlying this technique is an increase in density of interlobular septae resulting from transudate in the context of generalized fluid overload. This results in an acoustic mismatch between lung parenchyma and surrounding tissues, that causes partial reflection of the ultrasound beam. This creates discrete hyper-echoic reverberation artefacts arising from the pleural line known as “B-lines”.

We evaluated lung ultrasound in a single-centre pediatric study and found it to be a practical and sensitive method of quantifying fluid overload in children with dialysis-dependent AKI and ESKD [31]. Focused ultrasound examinations for B-lines take approximately 5–10 min to perform at the bedside and were well tolerated by infants and children. Quantification of B-lines on ultrasound related directly to fluid overload judged by acute increases in patient weight.

Further work is being undertaken to automate detection of B-lines on ultrasound, with the aim of increasing availability of this technique to patients and carers with limited training requirements [32].

### The next steps in optimizing assessment of fluid overload

Currently, traditional markers of fluid overload such as blood pressure, physical examination and inter-dialytic weight gain remain the main stay of fluid assessment in dialysis-dependent children. The emerging objective techniques discussed above have not yet been widely adopted in pediatric clinical practice. One reason for this may be the currently paucity of robust, multi-centre data evidencing their clinical benefit in children.

The choice of numerous techniques may also present a barrier to clinical adoption. We compared lung ultrasound, echocardiographic measurement of IVC parameters and bioimpedance spectroscopy in the assessment of fluid overload in children aged 0.8 to 14 years with ESKD [8]. Of the techniques evaluated in this study, lung ultrasound correlated best with fluid overload judged by acute increases in patient weight. Larger multi-centre studies are needed to confirm these findings.

Further work is also needed to improve the user friendliness of the above objective techniques for assessing fluid overload. While progress is being made in this regard [32, 33], more work is needed to increase usage by both health professionals and patients and parents/carers. When these barriers are overcome, objective assessment of fluid overload to enhance traditional clinical assessment is likely to become part of routine care for children on dialysis.

## Management of fluid overload

The clinical approach to managing volume overload should take into account the following evidence from adult and pediatric studies:

1. Higher IDWG is associated with higher blood pressure and LVMI in children on hemodialysis (HD) [34, 35];
2. Rapid ultrafiltration rates are associated with an increased risk of intradialytic morbidity, myocardial stunning and even increased mortality in adults on dialysis [5, 36, 37];
3. Missed target weights are associated with increased cardiovascular morbidity and mortality in adults with ESKD [38].

The only way to successfully manage fluid overload is therefore to control IDWG, ultrafiltration rates and postdialysis fluid overload simultaneously [39].

**Fig. 2:**
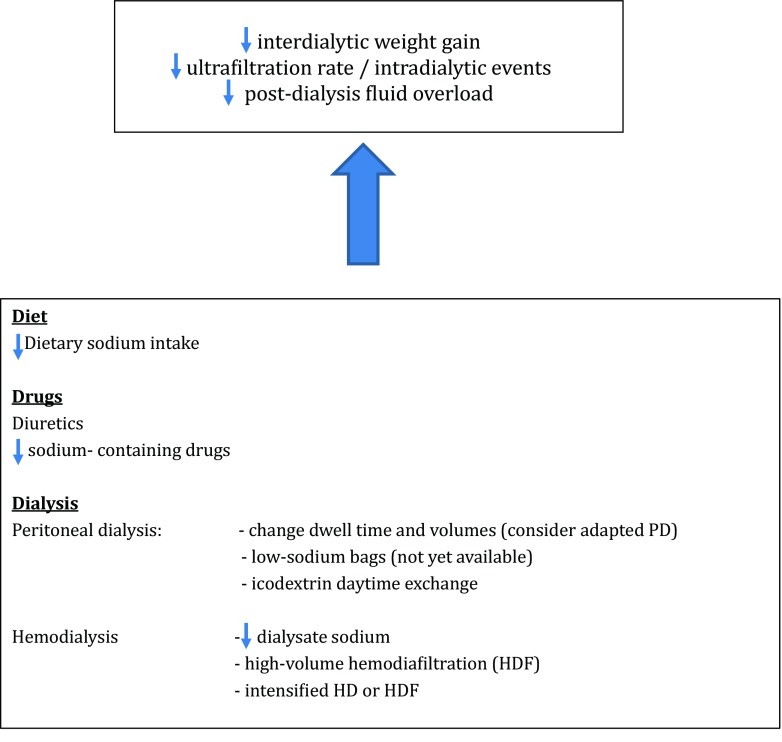
Summary of management strategies to minimize fluid overload. *PD* peritoneal dialysis, *HD* hemodialysis

As summarized in Fig. 2, a rational approach to fluid overload in children on maintenance dialysis should include reduction of dietary sodium intake and optimization of both medication and dialysis schedules.

### Reduction of dietary sodium intake

The rationale supporting dietary Na restriction in patients with ESKD is that they are primarily drinking in response to osmometric thirst, due to activation of osmoreceptor cells in the hypothalamus caused by increased extracellular fluid osmolality.

The benefits of a low-salt diet in patients with ESKD have been well demonstrated in adult studies [40–42]. Among them, a post-hoc analysis of 1770 patients in the hemodialysis study found that higher reported dietary sodium intake was independently associated with greater ultrafiltration requirements and greater all-cause mortality [40]. Two randomized crossover trials compared an Na-restricted diet versus a free diet in adults with 3–4 CKD: extracellular fluid volume, measured by bioimpedance spectroscopy, significantly decreased with sodium restriction in both studies, together with blood pressure and body weight [41, 42].

However, the possible detrimental effect of an overly restrictive diet on nutritional status needs to be born in mind.

The average dietary Na intake and its effect on fluid have never been investigated in children on dialysis. As regards predialysis children, data from the CKiD Study showed that the median Na intake in children with CKD 2–4 was 3089 mg/day, exceeding the recommended maximum daily intake for all age groups, in adolescents in particular [43]. Fast foods were the largest single source of sodium, contributing 9.4% of the total [44]. These data confirmed that pediatric patients usually enter ESKD without having adapted to salt restriction.

Both the Kidney Dialysis Outcome Initiative (KDOQI) and the Kidney Disease Improving Global Outcomes (KDIGO) guidelines recommend the restriction of Na intake for children with CKD who have hypertension or pre-hypertension, on the basis of the age-based recommended daily intake for healthy children [45, 46]: upper limits for Na intake are 1500 mg/day for children aged 2–3 years, 1900 mg/day for children aged 4–8 years, 2200 mg/day for children and adolescents aged 9–13 years and 2300 mg/day for the population aged ≥ 14 years.

But how can dietary Na restriction be achieved? Simply asking the parents not to add salt at the table or while cooking is not enough, as this source accounts for less than 15% of total Na intake, while salt added by manufacturers during food processing and Na occurring naturally in foods provide almost 75 and 10% of the total ingested Na, respectively. Continuous dietary counselling from an expert pediatric dietician is therefore mandatory to help children and their families to choose the right foods. The major obstacle to a low-salt diet in children is non-compliance, due to the common perception that low-salt foods are tasteless. When facing this problem, it should be remembered that sodium intake has all the characteristics of a true addiction, as salt sensing of the tongue is strictly dependent on the amount of ingested Na and adaptation of the taste receptors is a long process: only after several months of eating a low-salt diet will salt-rich food be perceived as too salty. Occasional salt-rich food intake hinders this process; therefore, the consumption of Na-rich food during dialysis should be discouraged. A major multidisciplinary effort is needed to help the child and their parents adapt to Na restriction.

### Medications

#### Diuretics

The role of diuretics in patients with ESKD and residual renal function is debated. Trials in adults on continuous ambulatory PD showed that diuretic use increases urinary water and sodium excretion, thus reducing IDWG, the need for aggressive ultrafiltration and the incidence of intradialytic hypotensive episodes, with a positive effect on residual renal function preservation and myocardial protection [47–49].

#### Sodium-containing drugs

Avoidance of Na-containing drugs can help minimize thirst in children with ESKD. Examples include Na bicarbonate (1 g contains 275 mg of Na) and sodium polystyrene powder (1 g contains 100 mg of Na). Data from the DOPPS showed that patients prescribed a Na-based exchange resin had greater IDWG and higher plasma Na than those who were not prescribed this treatment [50]. A recent randomized crossover trial on 20 predialysis adult patients with hyperkalemia showed that plasma Na and atrial natriuretic peptide were higher in patients treated with sodium polystyrene sulfonate than in those treated with calcium polystyrene sulfonate, suggesting that the latter can be safer in patients at risk of fluid overload [51].

### Optimization of PD prescription

#### Adaptation of PD exchanges

The mechanisms of fluid removal and transport during PD have traditionally been described by the 3-pore model [52]. According to this model, fluid transport is dependent on two opposing forces, the intraperitoneal osmotic pressure and the hydrostatic pressure gradient, the latter depending on the difference between intravascular and intraperitoneal hydrostatic pressure. As the osmotic agent during overnight PD exchanges is usually glucose, which is progressively reabsorbed during the dwell, the osmotic-driven free water transport through ultrasmall pores is highest during the first phase of a dwell; short dwells result in lower glucose reabsorption and improved total free water ultrafiltration. The intraperitoneal hydrostatic pressure is strictly dependent on dwell volume, with smaller fill volumes being associated with lower intraperitoneal pressure and higher ultrafiltration. Based on these principles, short dwells with low fill volumes are usually prescribed during standard automated PD to optimize fluid removal. With this PD schedule, a better ultrafiltration comes at the price of lower solute removal, Na in particular. Sodium removal during PD is mainly dependent on the Na concentration gradient between plasma and dialysate, integrity of the peritoneum, total peritoneal recruited area and time. Optimization of salt removal requires long dwells and high fill volumes.

To optimize both water and solute removal, the concept of adapted PD has been proposed, which comprises a few short dwell/small volume exchanges to improve ultrafiltration, followed by exchanges with a longer dwell time and larger fill volume to promote toxin and Na removal [53–55]. A multicentre prospective randomized crossover trial in 19 adults and a small crossover study in 4 children demonstrated that adapted PD resulted in higher ultrafiltration and Na removal compared with standard PD [54, 55]. These results were not confirmed in a computer simulation based on the 3-pore model, that showed a negligible benefit of adapted PD compared to conventional PD, emphasizing the importance of an accurate Na measurement and the need for larger trials [56].

#### Low-sodium PD fluids

The commercially available PD solutions contain 132 to 134 mmol/l of Na. Given that Na removal through diffusion is dependent on the transperitoneal concentration gradient, reducing the dialysate Na can be viewed as a strategy to increase Na removal. As sodium is osmotically active, low-Na PD solutions have beneficial effects on volume control only by simultaneously increasing the glucose concentration to maintain osmolality and ultrafiltration [57]. However, no such solutions are currently commercially available and pediatric data are sparse.

#### Icodextrin

Icodextrin is a solution of glucose polymers with average molecular weight 16,200 Da and pH 5.2. As only 45% of the infused icodextrin is absorbed at the end of a 14-h dwell, it can be safely used for the daytime exchange in patients on PD [58, 59]. Icodextrin fluid removal correlates significantly with age, which suggests that this solution could be particularly useful for older children [59]. In the largest study addressing ultrafiltration with icodextrin in children, Rousso et al. retrospectively reported their experience in 50 pediatric patients. A significant correlation was found between the daytime fill volume and ultrafiltration: in particular, a successful ultrafiltration was achieved in 88% of children with a fill volume above 550 ml/sqm body surface area versus 44% of those with a smaller fill volume [59].

Taken together, these studies suggest that icodextrin daytime exchange can safely be used to increase fluid removal, particularly in older children, and that a minimum icodextrin day dwell of 550 ml/sqm body surface area can facilitate ultrafiltration in pediatric patients maintained on PD.

### Optimization of HD prescription

#### Dialysate sodium

Sodium removal during dialysis is the sum of convective losses (with ultrafiltration) and diffusive losses, which are dependent on the diffusion gradient between plasma and dialysate. Under the usual dialysate Na prescription of 138–140 mEq/L, more than 80% of Na removal is convective. Reduction of dialysate Na has been proposed as a possible strategy to optimize Na and fluid management.

Some facts should be taken into account when considering this option:

- Measured dialysate Na is often not equivalent to prescribed dialysate Na and, in particular, a tendency towards higher measured dialysate Na than that prescribed has been reported [60]
- The availability of Na for diffusion across the HD membrane is influenced not only by plasma and dialysate Na concentrations, but also by the complexing of Na with other anions, by the Gibbs-Donnan effect and by Na concentration in plasma water, which is different from the total plasma concentration. It has been estimated that Na removal can occur only when dialysate Na is at least 2 mmHg lower than the plasma Na concentration.
- Lowering dialysate Na could be associated with lower thirst, lower IDWG and better blood pressure control, but a higher incidence of intradialytic events. On the contrary, patients treated with higher dialysate Na can tolerate the HD session better, but often at the price of higher IDWG and increased thirst and blood pressure levels [60, 61].
- The use of Na profiles has been proposed to optimize both HD tolerance and fluid control, but this strategy could be associated with high time-averaged Na concentrations, and high Na loading [60].

In summary, the available evidence does not allow for the identification of a “one size fits all” level of optimal dialysate Na [61]. In this context, it is not surprising that there is a discrepancy between the “Volume First” proposal put forward in the adult nephrology community, which emphasizes the need for avoiding intradialytic Na loading and suggests a dialysate Na of 134–138 mEq/L, and the position of the DOPPS Group, which suggests not prescribing dialysate Na concentration lower than 138 mEq/L [39, 62].

The pediatric experience in this field is much more limited. The huge intra- and inter-patient variability of pre-HD plasma Na has been confirmed in pediatric patients and makes the individualization of dialysate Na prescription challenging to implement in clinical practice. In a small pediatric study of 480 HD sessions in 5 children, a reduction of dialysate Na from 140 to 138 mEq/l was associated with lower IDWG and improved pre-HD systolic and diastolic blood pressure (from 133 to 127 and from 84 to 73 mmHg, respectively) [63].

Taking all these data into account, a reasonable approach could be to prescribe a dialysate Na of 138 mEq/l in most children treated with HD; lower dialysate Na should be considered in the case of severe difficulties in controlling IDWG and fluid excess, while prescription of higher dialysate Na should be restricted to children at high risk of intradialytic events.

#### Convective therapies

The benefits of convective therapies over standard bicarbonate HD have been investigated by some large randomized controlled trials and meta-analyses in adults, which showed that high-volume on-line hemodiafiltration (HDF), with a convective volume of at least 17–23 l/session in the post-dilution mode, is associated with improved overall survival compared to bicarbonate HD, mainly resulting from reduced cardiovascular mortality [64]. Different mechanisms have been advocated to explain these findings, including a lower incidence of intradialytic hypotension and better Na management with convective therapies.

Pediatric data comparing HD and HDF are still lacking. Preliminary data from the HDF-Heart-Height (3H) study demonstrated that prevalent pediatric patients treated with HDF were less likely to have fluid overload compared to those treated with bicarbonate HD according to bioimpedance spectroscopy. Although no difference in IDWG was observed, children on HDF required fewer rescue sessions [65].

Based on the available evidence, until the definitive results of the 3H study are published, high volume online HDF should be considered the dialysis modality of choice in children on extracorporeal dialysis at risk of fluid overload and cardiovascular impairment.

#### Intensified HD/HDF

In several adult clinical trials, intensified HD or HDF schedules (daily or nocturnal, home or in-centre HD or HDF) have been associated with clear clinical cardiovascular benefits, in particular, a lower need for aggressive ultrafiltration, lower IDWG, reduced intradialytic events and myocardial toxicity, better blood pressure control and reduced LVMI [66, 67]. Remarkably, a post hoc analysis carried out by the Frequent Hemodialysis Network trial showed that the improvement in left ventricular mass observed in cases of more frequent dialysis was likely caused by extracellular volume reduction directly and not only via an effect on blood pressure [67].

Some pediatric single-centre studies, which enrolled a total of more than 40 patients, confirmed the beneficial effect of daily and nocturnal HD or HDF on intermediate cardiovascular outcomes, such as blood pressure and LVMI [68–70].

Considering all the available evidence, intensified HD schedules may be considered the best strategy to counteract volume overload in patients with fluid-dependent cardiovascular impairment.

## Conclusions

Notwithstanding recent advances in the management of children with ESKD, adequate control of fluid remains an on-going clinical challenge. Both the assessment of target weight and body composition, and the management of fluid overload are still largely based on adult studies as limited pediatric data are available. Given the lack of a gold-standard method for body composition analysis, the prescription of target weight relies on a multi-parameter assessment, which can be enhanced with objective techniques including relative blood volume monitoring, bioimpedance analysis and lung ultrasound. The approach to fluid overload should include dietary counselling aimed at salt restriction, consideration of diuretics, minimizing sodium-containing drugs and optimization of the dialysis schedule, focusing on both sodium and fluid removal. Multi-centre pediatric studies are urgently needed to improve our knowledge in this field, in order to mitigate the short- and long-term cardiovascular sequelae of fluid overload in children on maintenance dialysis.

## Multiple choice questions (answers are provided following the reference list)

1. Physical examination of a 30 kg child by an experienced physician will detect signs of fluid overload when the level approaches:
  - 500 ml
  - 1 litres
  - 2 litres
  - 3 litres
2. In order to minimize fluid overload in children on hemodialysis, the ultrafiltration rate should be increased until:
  - Intradialytic symptoms occur
  - Intradialytic hypotension occurs
  - Myocardial stunning is evident on speckle tracking echocardiography
  - None of the above
3. In children on peritoneal dialysis, sodium clearance can be enhanced by the following measures:
  - Shortening the dwell time to short cycles
  - Addition of an icodextrin daytime exchange
  - Minimising sodium containing drugs
  - Lower glucose concentration dialysate
4. In a 2 year old child, inter-dialytic weight gain is affected by the following:
  - Dietary sodium intake
  - Nutritional prescription
  - Total daily fluid intake
  - All of the above
5. Which of the following statements are true:
  - NT-proBNP is a plasma marker of myocardial damage
  - Lung ultrasound detects generalized fluid overload via thickened interlobular septae
  - Multi-frequency bioimpedance spectroscopy estimates intravascular and extravascular water content
  - Blood volume transducers detect absolute changes in intravascular volume
